# A Repertoire of the Less Common Clinical Yeasts

**DOI:** 10.3390/jof9111099

**Published:** 2023-11-11

**Authors:** Estelle Menu, Quentin Filori, Jean-Charles Dufour, Stéphane Ranque, Coralie L’Ollivier

**Affiliations:** 1 Laboratoire de Parasitologie-Mycologie, IHU Méditerranée Infection, 13385 Marseille, France; stephane.ranque@ap-hm.fr (S.R.); coralie.lollivier@ap-hm.fr (C.L.); 2 Institut de Recherche pour le Développement, Assistance Publique-Hôpitaux de Marseille, Service de Santé des Armées, VITROME: Vecteurs-Infections Tropicales et Méditerranéennes, Aix Marseille Université, 13385 Marseille, France; 3 INSERM, IRD, SESSTIM, Sciences Economiques & Sociales de la Santé & Traitement de l’Information Médicale, ISSPAM, Aix Marseille University, 13385 Marseille, France; quentin.filori@hotmail.com (Q.F.); jean-charles.dufour@univ-amu.fr (J.-C.D.); 4 APHM, Hôpital de la Timone, Service Biostatistique et Technologies de l’Information et de la Communication, 13385 Marseille, France

**Keywords:** rare yeasts, uncommon yeasts, repertoire

## Abstract

Invasive fungal diseases are a public health problem. They affect a constantly increasing number of at-risk patients, and their incidence has risen in recent years. These opportunistic infections are mainly due to *Candida* sp. but less common or rare yeast infections should not be underestimated. These so-called “less common” yeasts include Ascomycota of the genera *Candida* (excluding the five major *Candida* species), *Magnusiomyces/Saprochaete*, *Malassezia*, and *Saccharomyces*, and Basidiomycota of the genera *Cryptococcus* (excluding the *Cryptococcus neoformans*/*gattii* complex members), *Rhodotorula*, and *Trichosporon*. The aim of this review is to (i) inventory the less common yeasts isolated in humans, (ii) provide details regarding the specific anatomical locations where they have been detected and the clinical characteristics of the resulting infections, and (iii) provide an update on yeast taxonomy. Of the total of 239,890 fungal taxa and their associated synonyms sourced from the MycoBank and NCBI Taxonomy databases, we successfully identified 192 yeasts, including 127 Ascomycota and 65 Basidiomycota. This repertoire allows us to highlight rare yeasts and their tropism for certain anatomical sites and will provide an additional tool for diagnostic management.

## 1. Introduction

Yeasts are unicellular eukaryotic organisms classified as members of the Kingdom Fungi. To date, more than 2000 species have been described, estimated to represent less than 1% of yeast species present on the Earth [1,2,3,4]. This number continues to grow with the widespread use of molecular taxonomy methods [5].

Yeast infections are among the top three healthcare-associated bloodstream infections in the United States, and they are in fourth place among all healthcare-associated infections, with *Candida* sp. leading the way [6]. With an attributable mortality rate of almost 40% in the case of systemic involvement, they represent a major public health issue [7]. Five species are responsible for ~90% of human fungal infections, namely, *Candida albicans*, *C. tropicalis*, *C. glabrata*, *C. parapsilosis*, and *Pichia kudriavzevii* (syn. *C. krusei*) [8,9]. Also, populations at risk of developing invasive fungal diseases are increasing, as are immunocompromised and severely ill patients [10], and the fungal repertoire is expanding to include less common or rare yeasts involved in human pathology [9,11]. The less common major pathogens include Ascomycota of the genera *Candida* (excluding the five major *Candida* species), *Magnusiomyces/Saprochaete*, *Malassezia*, and *Saccharomyces*, and Basidiomycota of the genera *Cryptococcus* (excluding the *Cryptococcus neoformans*/*gattii* complex members), *Rhodotorula*, and *Trichosporon*. Increasing attention is being paid to several emerging yeasts. One example is *Candida auris*, the involvement of which in human pathology has been increasingly reported since its first isolation in 2009, and the number of clinical cases rose from 329 in 2018 to 1012 in 2021 [12]. Others, such as *Pseudozyma* sp., although described inconsistently in the literature, may be responsible for systemic infections and interest in them should not be lost [13]. However, knowledge of the infections caused by these so-called “less common” or “rare” yeasts remains incomplete [10].

In this review, we offer an overview of less common or rare yeasts identified in humans as of 16 June 2020. Only publications reporting identification by culture and nucleotide analysis, whether or not they are associated with histopathology, were retained. We also provide information on the organs where these yeasts were isolated and on the semiology of the infections. As in our previous publication, our repertoire of the non-dermatophyte moulds [14] is divided into two. First, we describe the taxa of interest and their preferential site of infection, and then we describe which yeast species were involved at each major anatomical site.

## 2. Materials and Methods

### 2.1. Systematic Literature Review and Database Creation

We adopted the same approach to materials and methods as Menu et al. [14]. Briefly, all fungi names and synonyms were collected from both MycoBank (https://www.mycobank.org/ accessed on 15 November 2019) and NCBI Taxonomy (https://www.ncbi.nlm.nih.gov/taxonomy accessed on 15 November 2019). After aggregation and deduplication of these two listings, we obtained a single a list of 239,890 fungi taxa and any corresponding synonyms. For each fungus name in the list, a Python script version 3.7 and Biopython package version 1.74 [11] were used to query PubMed on 15 November 2019 to find bibliographical references that mentioned the fungus name or its synonyms, associated with the term “human” in the article title (TI), abstract (AB), author-supplied keywords (Other Term (OT)), or the Medical Subject Heading (MeSH) terms. The syntax of the queries was dynamically built using this pattern (fungi_name_or_synonyms [TIAB] OR fungi_name_or_synonyms [OT] OR fungi_name_or_synonyms [MeSH]) AND (“Human”[TIAB] OR “Human”[OT] OR “Humans”[MeSH]). A total of 7428 fungi taxa were found with at least one PubMed reference and incorporated in an MS Access^TM^ database (Access 2013, Microsoft, Redmond, WA, USA) created on 16 June 2020.

### 2.2. Manual Database Incrementation

As we previously described [14], the title and/or abstract and/or whole paper of each PubMed reference was manually analysed to ensure that it had been isolated from humans. As this process was laborious, a time limit of 16 June 2020 was set to ensure the same PubMed content for each fungal species. No start date limit was set.

**Figure 1:**
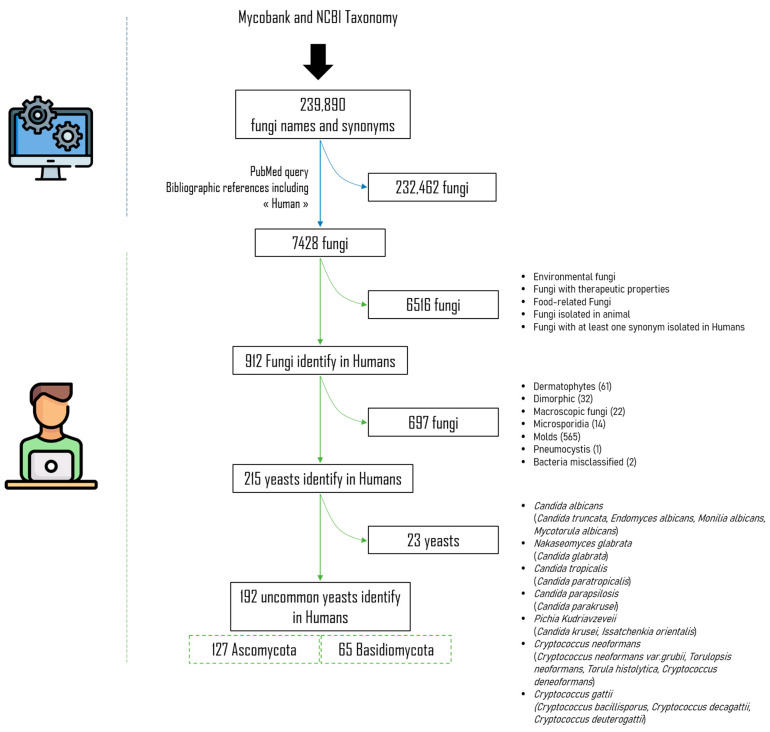
Systematic literature review flowchart.

After analysis, 6516 fungal taxa that were ultimately not found in humans were excluded. Filamentous fungi (moulds and dermatophytes), microsporidia, dimorphic fungi, and *Pneumocystis* isolated in humans were also excluded. We also excluded the most common yeasts and focused on the less common species. Among the Ascomycota, we therefore chose to exclude the five *Candida* species that are most commonly implicated in human diseases, namely, *Candida albicans*, *C. tropicalis*, *C. glabrata*, *C. parapsilosis*, and *Pichia kudriavzevii* (syn. *C. krusei*) [8,9]. Similarly, among Basidiomycota, we chose to exclude species of the *Cryptococcus neoformans* and *Cryptococcus gattii* complexes (Figure 1).

We analysed the titles and/or abstracts and/or full paper and/or Supplementary Materials, when available, of 192 uncommon yeast fungal names and synonyms isolated in humans to complete the information on the anatomical site involved and the associated infection semiology by filling in the PubMed Unique Identifier (PMID) of the publication concerned. Only identifications by direct diagnosis were taken into account, including culture (followed by morphological, Matrix-Assisted Laser Desorption Ionization Time of Flight (MALDI-TOF) mass spectrometry, and DNA sequence-based identification), whether or not this was associated with histopathological findings and Polymerase Chain Reaction (PCR). Publications reporting a species-level diagnosis based solely on histopathological examination or indirect methods were excluded. The date of first publication, last publication, and the name used were also reported in the software. The anatomical sites included have previously been detailed by Menu et al. [14].

### 2.3. Data Analysis

The MS Access^®^ database (Access 2013, Microsoft) was converted into two Excel files (Excel 2013, Microsoft). In the first file, the number of PMIDs per taxa was calculated by the anatomical site where the fungi were isolated. In the second, the number of PMIDs per fungus was given for the two main divisions (i.e., Ascomycota yeasts, Basidiomycota yeasts) and the subdivisions chosen by the authors correspond to the main yeast genera, namely, *Candida*, the *Magnusiomyces*/*Saprochaete* clade, *Malassezia*, and *Saccharomyces*, for the Ascomycota, and *Cryptococcus*, *Rhodotorula*, and *Trichosporon*, for the Basidiomycota, according to the infections associated with the isolation of the fungus, as reported by the authors. If more than one case was described in a publication with the same anatomical site, it counted for one publication due to a unique PMID.

### 2.4. Taxonomy

The taxonomy of yeasts has significantly evolved since the “one fungus, one name” nomenclature was adopted. We chose to divide the yeasts into Ascomycota and Basidiomycota. Within these major divisions, the subdivisions chosen by the authors correspond to the main genera, namely, *Candida*, the *Magnusiomyces*/*Saprochaete* clade, *Malassezia*, and *Saccharomyces* for the Ascomycota, and *Cryptococcus*, *Rhodotorula*, and *Trichosporon* for the Basidiomycota. We then used data from the literature to indicate the name of the species currently recommended, referred to as the “current name” [15,16]. We specified “Under classification” when the taxonomy had not yet been settled, as mentioned by Sugita et al. [16].

### 2.5. Synonyms

We referred to the “current name” in MycoBank and data in the literature to identify synonyms. The current name/synonym association was then checked by querying the PubMed database.

### 2.6. Figures

All figures were produced using the online tool WordArt version 4.17 (https://wordart.com/ accessed on 15 November 2019). The size of the name of each species was proportional to the number of times it occurred in the database. Ascomycota are represented in cold colours (*Candida* spp. in blue, Saccharomyces spp. in purple, the *Magnusiomyces*/*Saprochaete* clade in green, and others in grey-blue) and Basidiomycota in warm colours (*Cryptococcus* spp. In purple, *Rhodotorula* spp. In red, *Trichosporon* spp. In orange, and *Malassezia* spp. in pink).

## 3. Results

### 3.1. Fungal Location by Focusing on the Predominant Genus

In total, 2496 articles/PMIDs were included. This bibliographical research was divided in two groups respecting the two main divisions: Ascomycota and Basidiomycota. We identified 127 fungal species of 39 genera belonging to Ascomycota and 65 fungal species of 18 genera belonging to Basidiomycota, which had been reported at least once in humans.

#### 3.1.1. Ascomycota

The list of the Ascomycota taxa is detailed in Table S1 with their former and current scientific name, if applicable. Briefly, there were 80 *Candida* spp., 10 of the *Magnusiomyces*/*Saprochaete* clade, 5 *Saccharomyces* spp., and 33 other yeasts species distributed in 24 genera. Each of these taxa results are presented below. *Candida* was the leading genus; 1954 publications reported the isolation of these Ascomycota at every anatomical site (a publication could be counted multiple times due to the possible report of multiple anatomical sites in the same publication). This was followed by the *Saccharomyces* genus, followed by the *Magnusiomyces*/*Saprochaete* clade. For all Ascomycota, systemic localisation was the most common.

1. *Candida* spp.

Despite having excluded from our analysis the five *Candida* species responsible for over 90% of human infections, namely, *Candida albicans*, *C. tropicalis*, *C. glabrata*, *C. parapsilosis*, and *Pichia kudriavzevii* (syn. *C. krusei*), the other *Candida* species were the most frequently isolated yeasts in human clinical samples [8,9]. Two species were strongly represented: *Candida dubliniensis* and *Candida auris*. In terms of the time lapse between first description and last publication, *Candida auris* eclipses *Candida dubliniensis* (11 years and 25 years, respectively). These species emerged since their first description in 1995 in oral candidiasis in an HIV patient and from the external ear in 2009 in a patient treated at a Japanese hospital [17,18], respectively. The number of publications for *Candida dubliniensis* in human pathologies is probably underestimated because it had been commonly identified as *Candida albicans* before 1995 [19]. This is followed by *Candida lusitaniae* and *Candida guilliermondii* (syn. *Meyerozyma guilliermondii*). Although *Candida* is one of the most commonly found species in the normal human microbiota, it can become an opportunistic pathogen in the presence of risk factors [20,21]. The spectrum of candidiasis is broad, ranging from cutaneous to systemic candidiasis [22]. In this repertoire, *Candida* were mainly isolated from the systemic level with a total of 669 publications. The next anatomical sites were the oto-rhino-laryngology (ORL) system (279 publications, of which 130 concerned *C. dubliniensis*), the urinary tract, and the skin system, probably linked to the mucosal commensal nature of *Candida* species. In less well-represented anatomical sites, such as the skeletal system, we find a majority of *Candida auris* species, followed by *Candida lusitaniae*, *Candida guilliermondii*, and *Candida dubliniensis*. The same four species were also found in the central nervous system. Interestingly, *Candida auris* was mainly reported from post-neurosurgery ventriculitis [23,24,25] while *Candida dubliniensis* was reported from meningitis secondary to haematogenous dissemination [26,27,28,29], suggesting a potential species-dependent invasiveness not to be overlooked. Finally, *Kodamaea ohmeri* (formerly *Pichia ohmeri*) stands out with a total of 56 publications. This yeast, used in the food industry for fermentation, is commonly isolated from the environment [30] and was isolated in the majority of cases at the systemic level (31 publications).

2. *Magnusiomyces/Saprochaete* clade

The four species involved in human pathology forming the *Magnusiomyces/Saprochaete* clade are *Magnusiomyces clavatus*, *Geotrichum candidum*, and *Magnusiomyces capitatus*. The taxonomy has been revised many times [31]. These arthroconidia yeasts present in the environment are often responsible for opportunistic breakthrough infections in severely immunocompromised populations [32]. Systemic involvement stands out (95 publications), which is consistent with the literature, as bloodstream infections with or without pulmonary infections or skin lesions are the most frequent in at-risk patients [32,33]. The lung, gut, skin, ORL system, and urinary system also stand out, which may correspond to the ability of these yeasts to colonise these human anatomical sites [10].

3. *Saccharomyces* spp.

Comprising only three species found in human pathology, *Saccharomyces* spp. represented a total of 321 publications. *Saccharomyces cerevisiae*, commonly known as brewer’s or baker’s yeast due to its wide use in the food industry, is the leading cause of bloodstream infections (91 publications) [34]. These infections often occur secondarily to the use of *Saccharomyces boulardii* orally administered probiotics in immunocompromised patients [35]. They are infrequently commensal of mucosal surfaces such as the gastro-intestinal tract, which explains its isolation within it [36]. This repertoire highlights the isolation of *S. cerevisiae* from the genitalia and ORL system.

4. Others

Among the ascomycetous yeasts that were not classified within the previous three genera, *Lodderomyces elongisporus* stands out, with a total of ten publications. This rare yeast has mostly been isolated from bloodstream infections. Cardiac involvement has also been reported in the form of native valve endocarditis in an intravenous drug user, emphasising its potential invasiveness [37]. *Pichia farinosa* and *Sporopachydermia cereana* were found in systemic locations. *Torulopsis magnoliae* was isolated from the eye and was involved in endophthalmitis following cataract surgery [38].

#### 3.1.2. Basidiomycota

After excluding the *Cryptococcus neoformans* species complex and *Cryptococcus gattii* species complex, the list of the Basidiomycota taxa is presented in Table S2 with their former and current scientific names, if applicable. Briefly, there were 16 *Cryptococcus* spp., 13 *Malassezia* spp., 7 *Rhodotorula* spp., 19 *Trichosporon* spp., and 11 other species distributed in six genera. Each of these taxa results is presented below. *Trichosporon* was the leading genus, with 809 reports of the isolation of these Basidiomycota at almost all anatomical sites. In second place were the *Malassezia* yeasts, followed by the *Rhodotorula* and uncommon *Cryptococcus* species. For all Basidiomycota, cutaneous involvement is the most common (525 publications), with *Malassezia* accounting for 60% of the publications. Systemic localisation ranks second, with 398 publications in total; but obviously for species belonging to the genera *Cryptococcus*, *Rhodotorula*, and *Trichosporon*, the skin remains the most common location.

1. *Cryptococcus* spp.

*Cryptococcus* species are basidiomycetous encapsulate yeasts, ubiquitous in the environment [39]. In this repertoire, we chose not to analyse species belonging to the *Cryptococcus neoformans* complex and *Cryptococcus gattii* complex in order to focus on uncommon *Cryptococcus* species. We found 14 species isolated in humans, with a predominance of *Papiliotrema laurentii* (formerly *Cryptococcus laurentii*) and *Naganishia albida* (formerly *Cryptococcus albidus*). Compared to *Cryptococcus neoformans*, we found a tropism of these both species for the bloodstream (19 and 13 publications, respectively), the lungs (7 publications each), the cutaneous system (9 and 12 publications, respectively), and the central nervous system (8 and 3 publications, respectively), also reported in the literature [40]. Two species were isolated from the osteo-articular system: *Cryptococcus albidus* without detail of its location [41] and *Cryptococcus luteolus* from a case of tenosynovitis [42]. Interestingly, ocular involvement was reported for *Cryptococcus albidus*, *Cryptococcus laurentii*, and *Cryptococcus curvatus*, and this was mostly of the keratitis type. No cardiac, hepatic, skeletal, endocrinal, placental, or dental involvement was reported.

2. *Malassezia* spp.

*Malassezia* spp. are known to be major human skin commensals with pathogenicity limited to the skin [43]. This is reflected in our repertoire, with cutaneous localisation predominating with 316 publications of the 525 in total (60%). These Basidiomycota also inhabit the mucosal sites of humans, as shown by the ORL system, the digestive tract, and the genital localisation (13 publications each). However, among the *Malassezia* genus, three species have been isolated from bloodstream infections: *Malassezia furfur*, *Malassezia pachydermatis*, and *Malassezia sympodialis*, highlighting its potential invasiveness. *Malassezia* infections are probably underestimated due to their dependence on lipids, which makes its isolation by culture complicated in the absence of specific media [44]. Interestingly, 13 publications reported the isolation of *Malassezia* from the eye, with a majority with periocular involvement, namely, blepharitis and dacryocystitis, and a rare case of *Malassezia restricta* keratitis [45].

3. *Rhodotorula* spp.

*Rhodotorula* spp. are widespread in the environment and are commonly part of the intestinal mycobiome [46]. In this repertoire, we found five species isolated in humans. *Rhodotorula mucilaginosa* (formerly *Rhodotorula rubra*) was the predominant species with a total of 105 publications, followed by *Rhodotorula glutinis* with a total of 23 publications and *Cystobasidium minutum* (formerly *Rhodotorula minuta*) with a total of 10 publications. These three species were mainly responsible for fungaemia in immunocompromised patients and were therefore isolated at the systemic level [47]. Interestingly, *Rhodotorula mucilaginosa* was isolated in the skin system (17 publications), responsible for superficial cutaneous involvement. It was the only *Rhodotorula* species responsible for onychomycosis [48,49,50,51,52], demonstrating a capability to degrade keratin, as demonstrated in the literature [52]. One publication reported liver involvement secondary to pulmonary infection, pointing to the pathogenicity of this species [53]. Interestingly, *Rhodotorula toruloides* was isolated once from the bloodstream, and the unique case of *Rhodotorula pilimanae* in humans was a native infective endocarditis.

4. *Trichosporon* spp.

*Trichosporon* is widely represented in our repertoire. We found 19 species comprising two largely predominant species, *Cutaneotrichosporon cutaneum* (formerly *Trichosporon beigelii*, *Trichosporon cutaneum*, and *Trichosporon cutaneum*) and *Trichosporon asahii*. These species are found in an equivalent way in almost all locations except for the teeth, gums, and placenta. The two main localisations are systemic (90 and 86 publications, respectively) and cutaneous (55 publications for each), reflecting the vast pathogenic power of *Trichosporon*, from superficial skin damage known as white piedra to blood-borne dissemination occurring mostly in immunocompromised patients [47]. Our attention was drawn to the cardiac tropism of *Cutaneotrichosporon cutaneum* (26 publications), resulting in various types of infections, such as endocarditis mostly on prosthetic valves [54], and pericardium or myocardium involvement in disseminated cases [55,56]. Three other species appear to share this particular cardiac tropism, *Trichosporon asahii*, *Trichosporon inkin*, and *Trichosporon mycotoxinivorans*, which should not be ignored.

5. Others

Among the Basidiomycota not classified in these four genera, *Pseudozyma aphidis* stands out, with a total of seven publications. This opportunistic pathogen has been described as an environmental yeast and is found at the systemic level, pulmonary level, and cutaneous level. As it has also not yet been isolated in the human digestive tract, gastro-intestinal translocation is presented as a probable source of infection [57]. Another rare fungus, *Sporobolomyces salmonicolor*, stands out for its isolation being limited solely to deep sites with systemic involvement, endophthalmitis-like ocular involvement, and two meningitis-like central nervous system involvements [58,59,60,61,62]. Likewise, the only reported isolation of *Sporobolomyces roseus* was from the cerebrospinal fluid of a patient with meningitis [63].

### 3.2. Fungal Location by Focusing on Anatomical Site

Within the 19 anatomical sites, the semiology of infection was detailed for the five major categories of fungi involved in human pathologies (Table 1).

#### 3.2.1. Systemic

The systemic involvement is the anatomical site most affected by fungal yeast infections.

**Figure 2:**
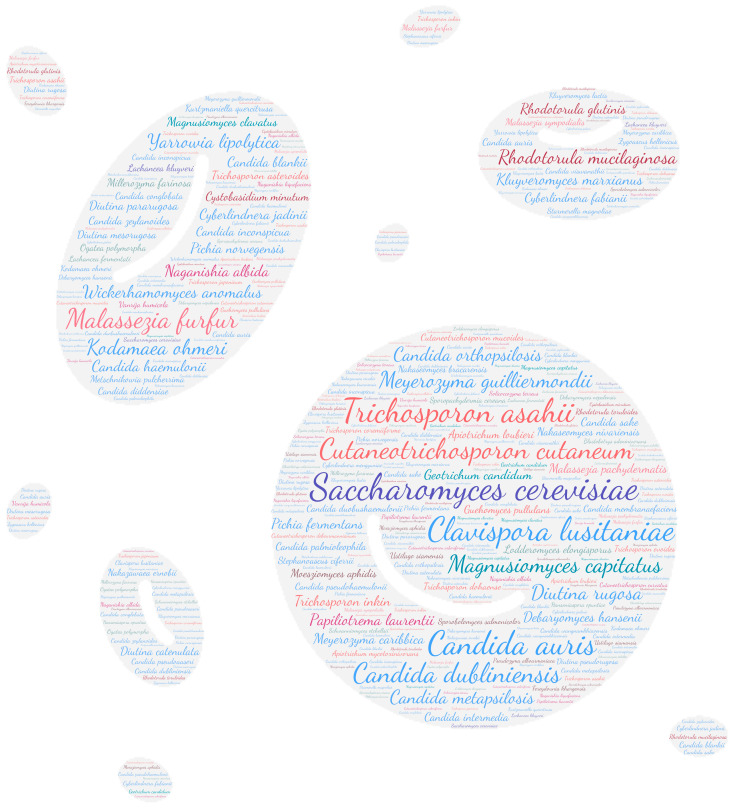
WordCloud of the yeast species involved in systemic infections. The size of the name of each species is proportional to the number of times it occurs in the repertoire.

Regarding systemic involvement (including fungaemia, aortitis, vasculitis, lymph node infection, and bone marrow infection), there is a large majority of fungaemia, especially candidaemia. Almost all yeasts can cause fungaemia (Figure 2). *Saccharomyces cerevisiae*, *Cutaneotrichosporon cutaneum*, *Candida auris,* and *Trichosporon asahii* are the predominant species, with over 85 publications each. These emerging species are nosocomial opportunistic pathogens [64,65]. In this review, 41% (35/86) of *Candida auris* isolated in the bloodstream was related to positive catheter culture, which a key risk factor due to the potential for biofilm formation [65].

*Saccharomyces cerevisiae* is the leading pathogen, with 91 publications (synonyms included). This yeast, widely used in the food industry in the fermentation process, can be used as a probiotic to modulate the digestive microbiota. Unfortunately, its use as probiotic is dangerous in severely immunosuppressed patients and can lead to fungaemia [66].

#### 3.2.2. Central Nervous System

In most cases, damage to the central nervous system results in meningitis (54 publications) and, rarely, in meningo-encephalitis (10 publications) or brain abscess (10 publications). A single case of encephalitis was reported due to *Geotrichum capitatum* (new *Magnusiomyces capitatus*) infection [67].

**Figure 3:**
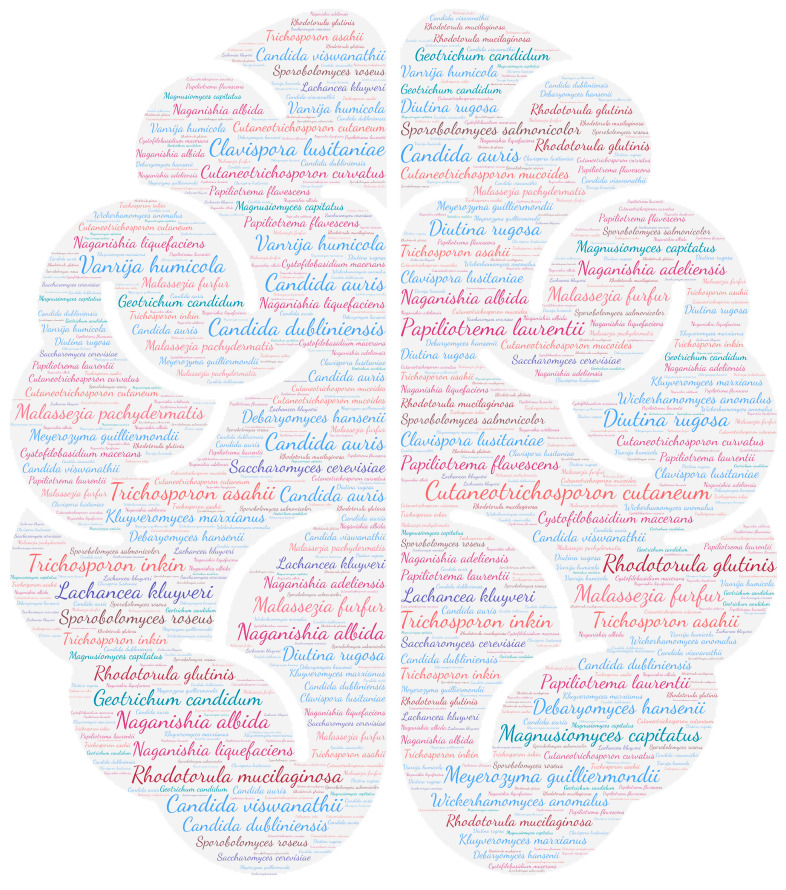
WordCloud of the yeast species isolated from the central nervous system. The size of the name of each species is proportional to the number of times it occurs in the repertoire.

The taxa *Candida* spp., followed in order by *Trichosporon* spp., *Cryptococcus* spp., and *Rhodotorula* spp., was predominant (Figure 3). The prevalence of *Rhodotorula mucilaginosa* and *Rhodotorula glutinis* in the CNS underlines the invasive potential of these ubiquitous yeasts, which are widespread in the environment. Also known to be less virulent than *Cryptococcus* sp. or *Candida* sp., they may be responsible for meningitis or ventriculitis in immunosuppressed individuals [68].

On the species level, *Cutaneotrichosporon cutaneum* and *Trichosporon asahii* were predominant, followed by *Candida dubliniensis*, *Papiliotrema laurentii,* and *Rhodotorula mucilaginosa* (Table S1).

#### 3.2.3. Eye

**Figure 4:**
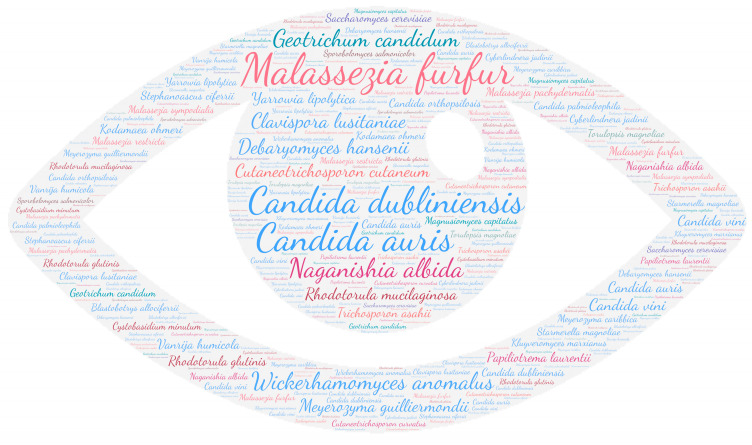
WordCloud of the yeast species isolated from the eye. The size of the name of each species is proportional to the number of times it occurs in the repertoire.

All categories of fungi can affect the ocular system. However, in this review, the *Candida* spp. taxa stands out (Figure 4). Ocular diseases due to *Candida* sp. can manifest as endophthalmitis or keratitis. Endogenous endophthalmitis was frequently encountered secondary to a bloodstream infection [69,70]. Fungal keratitis mainly developed secondary to keratoplasty [71,72,73,74], trauma [75,76,77], or immunosuppressive therapy [78]. As species level, *Malassezia furfur* was predominant with *Candida dubliniensis*, *Wickerhamomyces anomalus,* and *Candida auris* (Table S1).

*Malassezia* spp. were mainly isolated in periocular sites causing blepharitis, dacryocystitis, or conjunctivitis (Table 1). We found a rare case of *Malassezia restricta* keratitis secondary to soil contamination in a farm worker [45].

Surprisingly, less common *Cryptococcus* species were described in ocular involvement (nine publications) responsible for keratitis related to contact lens wear [79,80], post-surgery [81], or following plant trauma [82]. These are the species *Cutaneotrichosporon curvatus*, *Naganishia albida,* and *Papiliotrema laurentii* and synonyms.

#### 3.2.4. Auditory System

**Figure 5:**
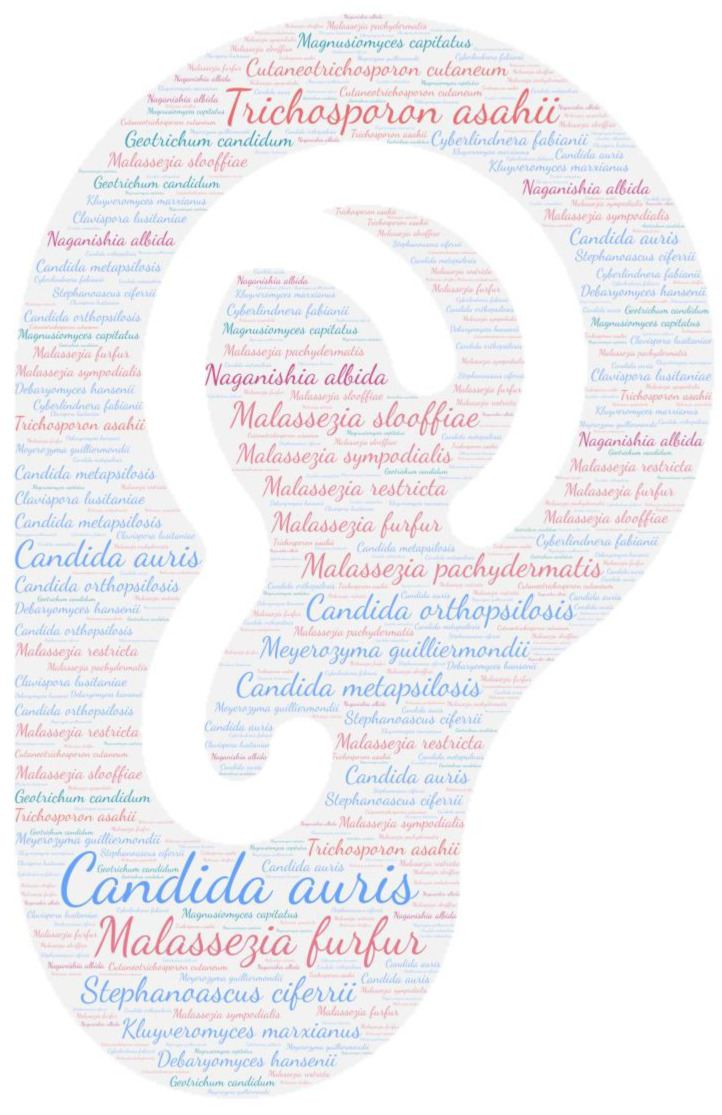
WordCloud of the yeast species name isolated from the ears. The size of the name of each species is proportional to the number of times it occurs in the repertoire.

As stated by Bojanović et al. [83], species of the genus *Candida* are predominant (Figure 5). Among the uncommon species, *Candida auris* is by far the majority, with 21 publications reporting it being isolated from the auditory system. It has a well-known tropism for the auditory system, as it was firstly isolated from the external ear in 2009 in a hospital in Japan [18]. Fewer cases report the isolation of *Malassezia* spp. from the auditory system, as in most cases it is part of the microbiota of the external ear canal [84]. However, one case of malignant otitis externa with *Malassezia sympodialis* in a diabetic patient has been reported, highlighting a potential pathogenic effect [85]. Similarly, *Trichosporon* species have more rarely been isolated from the auditory system and implicated in otomycoses (Table 1).

Among the rarely represented genera, we found a unique case report of *Geotrichum candidum* isolation in otitis externa in Turkey [86]. Among the genus *Cryptococcus*, only one case of otomycosis due to *Naganishia albida* was reported with manipulation of the ear canal considered as a risk factor [87].

#### 3.2.5. Oto-Rhino-Laryngology System

**Figure 6:**
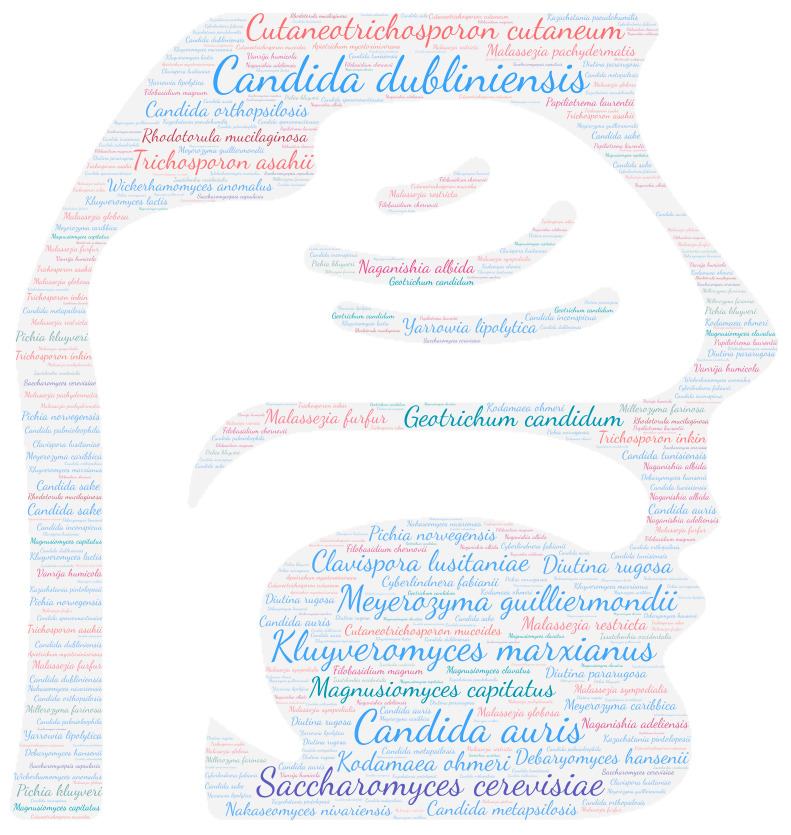
WordCloud of the yeast species isolated from the oto-rhino-laryngology system. The size of the name of each species is proportional to the number of times it occurs in the repertoire.

We found a majority of Ascomycota in the ORL system with 79% of *Candida* species (279/351) (Table S1; Figure 6). The predominant anatomical site was the oral mucosa, which is not surprising, as the *Candida* genus is known to colonise the oral cavity [88]. Yeasts then have the ability to go from a commensal to a pathogenic state in the presence of risk factors such as immunosuppression. The species most commonly implicated in oral candidiasis are those of the *Candida* genus, followed by species of the *Saccharomyces* genus [89].

#### 3.2.6. Pulmonary System

**Figure 7:**
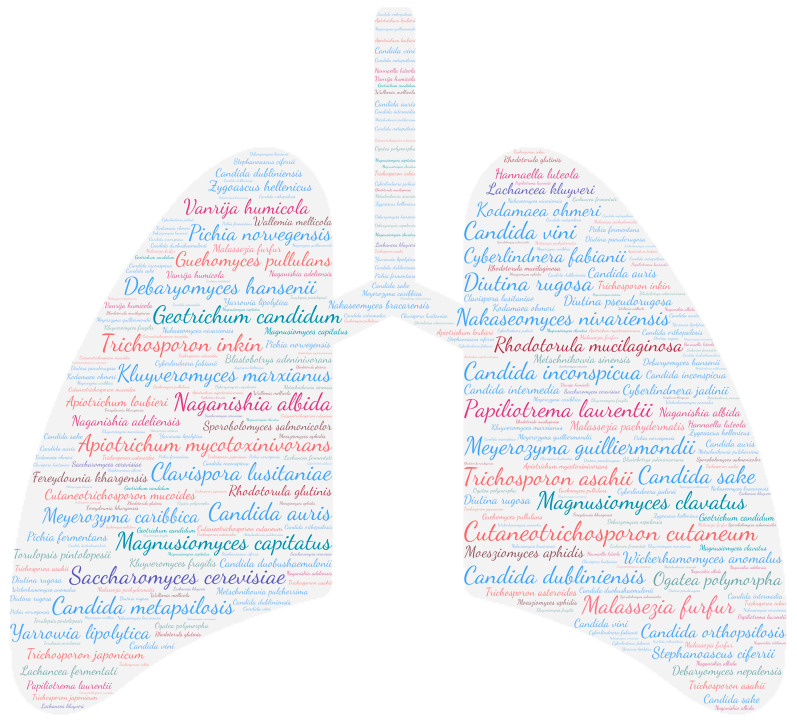
WordCloud of the yeast species isolated from the pulmonary system. The size of the name of each species is proportional to the number of times it occurs in the repertoire.

All the different taxa for both Ascomycota and Basidiomycota are represented in the pulmonary system (Figure 7). We found a majority of *Candida* species and *Trichosporon* species. Colonisation of the upper and lower respiratory system is the prerequisite for an invasive infection. Real lung infections remain rare and include yeast pneumonia (49 publications), lung abscess (2 publications), and the lung cavity (4 publications). Among the species responsible for yeast pneumonia, two stand out, namely *Cutaneotrichosporon cutaneum* and *Magnusiomyces capitatus*. Other species may be encountered on an anecdotal basis with a single publication, such as *Malassezia pachydermatis*, *Rhodotorula glutinis*, *Sporobolomyces salmonicolor*, *Cyberlindnera jadinii*, *Lachancea kluyveri*, *Naganishia adeliensis*, *Trichosporon asteroides*, *Metschnikowia pulcherrima*, *Candida duobushaemulonii*, *Pichia fermentans*, *Candida intermedia*, *Apiotrichum loubieri*, *Nakaseomyces bracarensis*, *Candida sake*, *Lachancea fermentati*, *Diutina pseudorugosa*, *Zygoascus hellenicus*, *Blastobotrys adeninivorans*, *Debaryomyces nepalensis*, *Fereydounia khargensis*, *Wallemia mellicola*, *Hannaella luteola*, *Kluyveromyces fragilis*, *Metschnikowia sinensis*, and *Torulopsis pintolopesii*.

The lower respiratory tract is the most frequent localisation (186 publications) followed by the upper respiratory tract (182 publications).

#### 3.2.7. Cardiac Involvement

**Figure 8:**
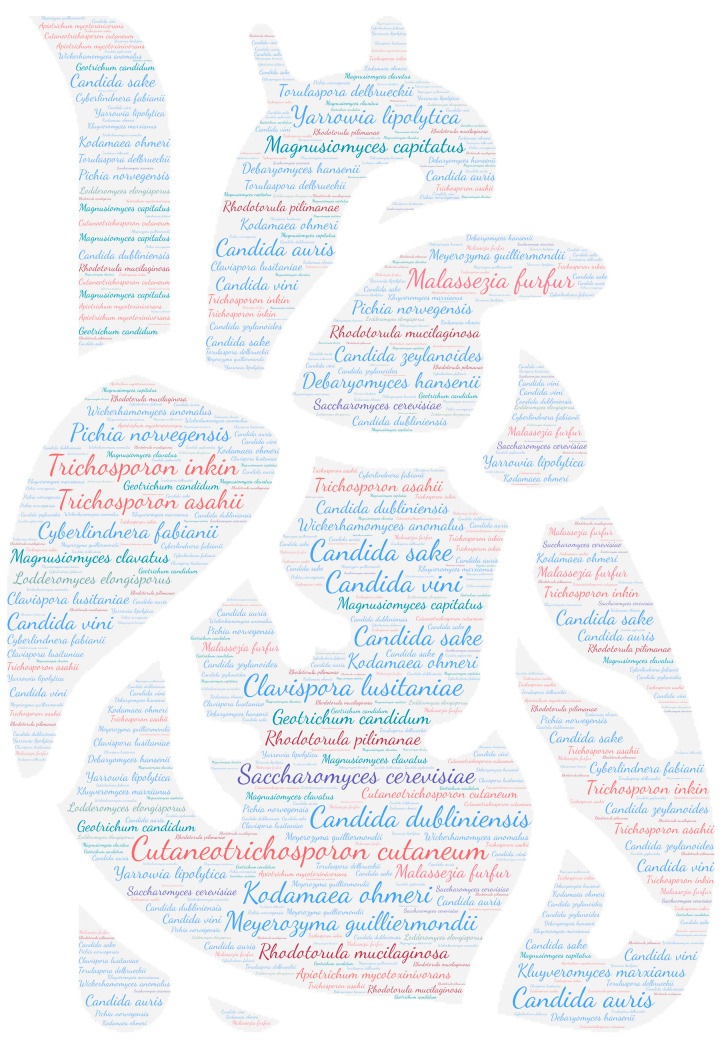
WordCloud of the yeast species isolated from the heart. The size of the name of each species is proportional to the number of times it occurs in the repertoire.

Cardiac involvement was found in 12% (23 species/192) of the less common yeast species described in this repertoire. All the taxa belonging to the Ascomycota were represented in this localisation and were responsible for native or prosthetic valve endocarditis. Interestingly, among the Basidiomycota, only the taxa *Trichosporon* seems to have a cardiac tropism and, more rarely, *Rhodotorula* and *Malassezia*. *Cutaneotrichosporon cutaneum* (formerly *Trichosporon beigelii* and *Trichosporon cutaneum*) was the main species involved in this localisation (26 publications) with three other *Trichosporon* species, namely, *Trichosporon asahii*, *Trichosporon inkin,* and *Trichosporon mycotoxinivorans* (Figure 8). Cardiac involvement was mainly in the form of native valve endocarditis (23 publications) followed by prosthetic valve or implanted equipment endocarditis (15 publications). Focusing specifically on the sites concerned, the aortic valve (11 publications), mitral valve (10 publications), and tricuspid valve (7 publications) were the most frequently concerned (Table 2).

#### 3.2.8. Digestive System

Concerning the digestive system, yeasts are mostly isolated from the peritoneum and implicated in peritonitis. In this review, 82% (61/74) of the publications reported peritoneal dialysis-related peritonitis. When data were available, other risk factors for peritonitis were underlying neoplasia [118], gastric and duodenal ulcer perforation [119], recent digestive surgery [120,121,122,123,124], severe immunosuppression [125,126], and pancreatitis [127]. In some cases, infection of the peritoneum, as well as other unusual infection sites including the biliary tract (9 publications), gastric tract (12 publications), and spleen (19 publications), may also be a secondary condition to the haematogenous dissemination of yeasts [128].

**Figure 9:**
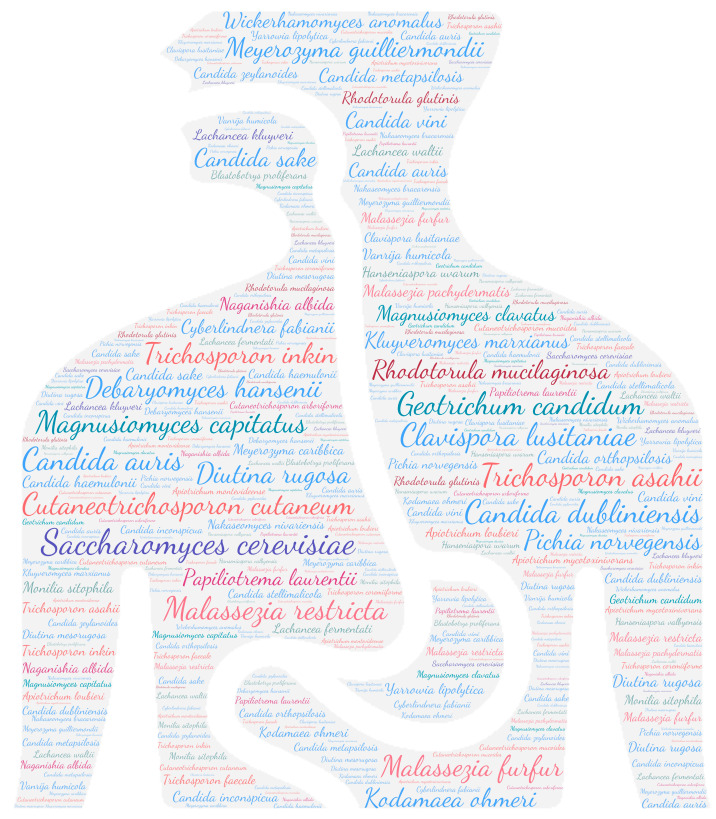
WordCloud of the yeast species isolated from the gut. The size of the name of each species is proportional to the number of times it occurs in the repertoire.

The species mostly found were *Saccharomyces cerevisiae*, *Cutaneotrichosporon cutaneum*, *Candida auris*, *Clavispora lusitaniae*, *Magnusiomyces capitatus*, *Trichosporon asahii*, *Geotrichum candidum*, and *Meyerozyma guilliermondii* (Table S1; Figure 9).

#### 3.2.9. Liver Involvement

**Figure 10:**
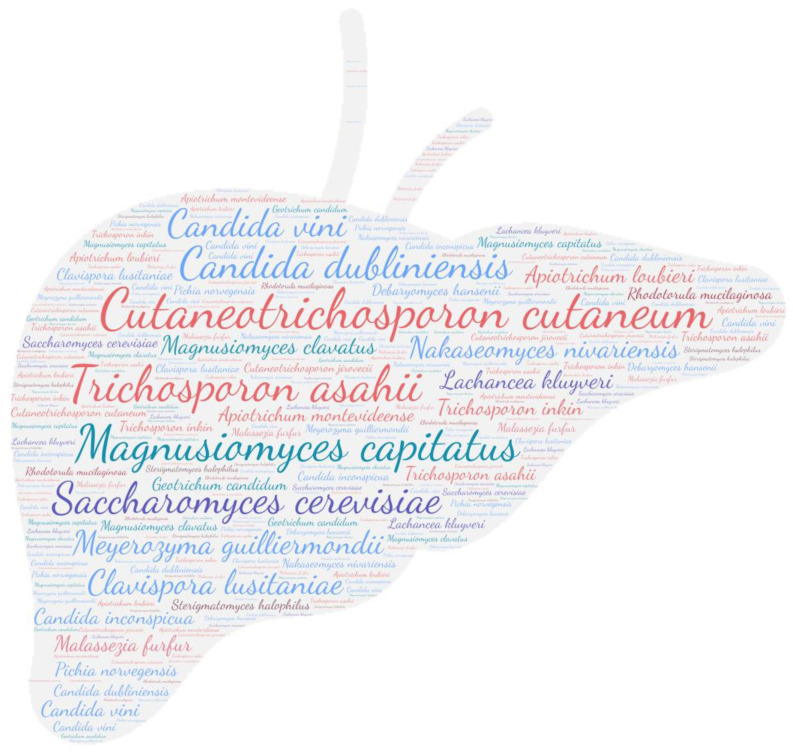
WordCloud of the yeast species isolated from the liver. The size of the name of each species is proportional to the number of times it occurs in the repertoire.

Liver involvement was predominantly described among the taxa *Trichosporon* and *Candida*, resulting in ascites, abscesses, and hepatitis (Figure 10). Less frequently, cases have also been reported for the *Magnusiomyces*/*Saprochaete* clade (nine publications); *Saccharomyces* spp. (seven publications), mainly *Saccharomyces cerevisiae*; *Malassezia furfur* (six publications); and *Rhodotorula mucilaginosa* (one publication). Interestingly, the only publication reporting isolation of *Sterigmatomyces halophilus* was a case of a liver abscess in an immunocompromised patient due to a marine-derived Basidiomycota [129].

#### 3.2.10. Urinary Tract

**Figure 11:**
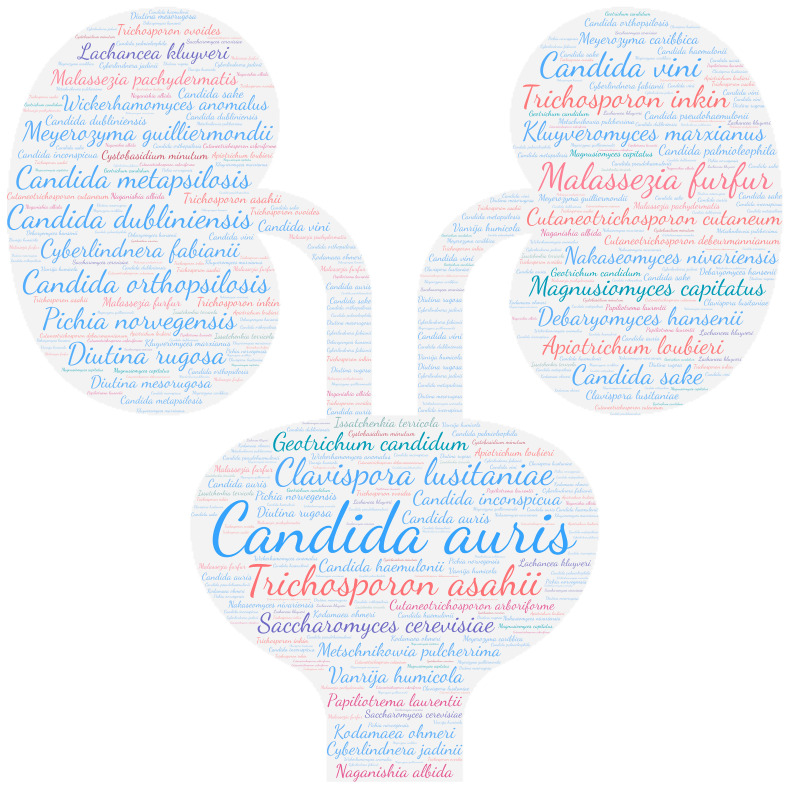
WordCloud of the yeast species isolated from the urinary tract. The size of the name of each species is proportional to the number of times it occurs in the repertoire.

In the urinary tract, yeasts were most frequently isolated in urine samples with a clear predominance of species of genus *Candida* (62%; 162/263) (Figure 11). Funguria are mainly nosocomial infections and urinary catheterisation plays an important role in urinary tract infections due to biofilm formation [130,131,132]. Unfortunately, due to a lack of data concerning the presence or otherwise of a urinary catheter, we were not able to assess the proportion of catheter infections in this review.

*Candida auris* was the most isolated species followed closely by *Trichosporon asahii*. *Trichosporon asahii* is known to be the most frequent pathogen causing urinary tract infections among the genus *Trichosporon.* This site of infection is considered as “uncommon” [133]. *Clavispora lusitaniae*, *Cutaneotrichosporon cutaneum*, and *Candida dubliniensis* were also represented.

#### 3.2.11. Osteo-Articular System

**Figure 12:**
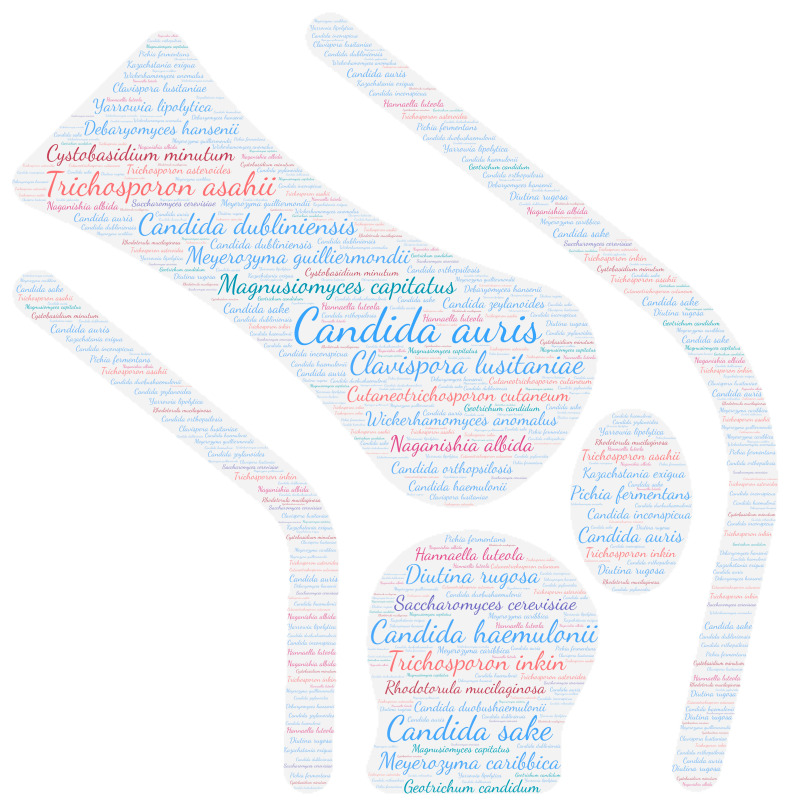
WordCloud of the yeast species isolated from the osteo-articular system. The size of the name of each species is proportional to the number of times it occurs in the repertoire.

Among the osteo-articular diseases, yeasts can cause osteomyelitis (17 publications) as well as arthritis (14 publications) and, more rarely, spondylodiscitis (9 publications). *Candida* species are predominant, followed by *Trichosporon* species (Figure 12).

Four *Rhodotorula mucilaginosa* osteo-articular diseases were reported, one post-operative persistent femoral non-union [134], one femoral prosthesis infection [135], one hip-joint prosthesis infection [136], and one case of an infection associated with multifocal skeletal tuberculosis [137]. *Saccharomyces cerevisiae* was responsible for two cases of osteomyelitis [138,139] and *Cryptococcus luteolus* was responsible for a case of tenosynovitis [42].

#### 3.2.12. Skin System

**Figure 13:**
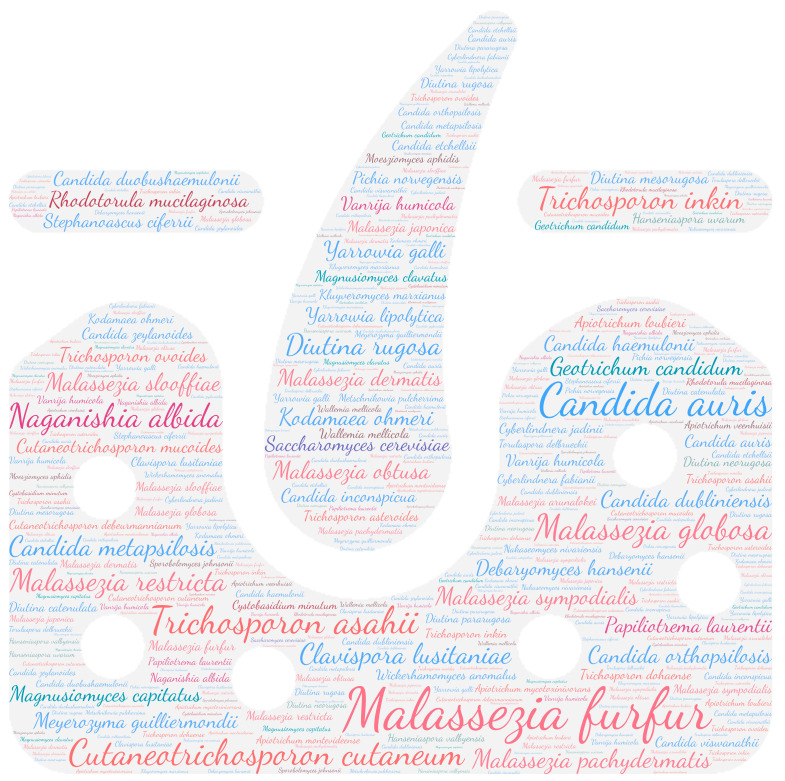
WordCloud of the yeast species isolated from the skin system. The size of the name of each species is proportional to the number of times it occurs in the repertoire.

Basidiomycota are predominant in cutaneous system involvement (587 publications), with *Cryptococcus* species as the leader (Figure 13). Cryptococcal skin lesions may be primary cutaneous lesions or sentinels for disseminated disease [140]. The second leading yeasts were *Candida* species. These Ascomycota may be a commensal on the skin but can also become pathogenic in the presence of predisposing factors such as impaired immunological status or skin barrier disruption [22,141]. However, deep cutaneous *Candida* infections are uncommon [142], as subcutaneous infections are rarely due to *Candida auris* [143,144], *Candida duobushaemulonii* [142], or *Candida rugosa* [145]. *Trichosporon* species appeared in third place. These too are part of the normal skin microbiota but can cause a common superficial infection in tropical and subtropical regions known as “the white piedra” [47,146].

In this repertoire, it is thus not surprising that we found a majority of superficial cutaneous lesions, followed distantly by onychomycosis (Table 1). A few subcutaneous involvements were described, caused by *Candida auris*, *Candida duobushaemulonii*, *Candida rugosa*, *Papiliotrema laurentii*, *Cutaneotrichosporon debeurmannianum*, *Kodamaea ohmeri*, *Magnusiomyces capitatus*, *Malassezia furfur*, *Pichia ohmeri*, *Saccharomyces cerevisiae*, *Trichosporon asahii*, *Trichosporon cutaneum*, *Trichosporon inkin*, *Trichosporon montevideense*, *Trichosporon ovoides*, and *Wallemia sebi*. Two yeasts, *Candida guilliermondii* and *Pseudozyma aphidis*, were isolated from mycetoma, but they were associated with the isolation of *Madurella mycetomatis* and *Nocardia otitidiscaviarum*, respectively [147,148].

#### 3.2.13. Genital Sphere

Genital sphere involvement is mostly related to the *Candida* genera and *Saccharomyces* genera (Table S1) [130]. *Saccharomyces cerevisiae* is the leading species, followed by *Candida dubliniensis*, *Clavispora lusitaniae*, and *Meyerozymaguilliermondii*.

Genital sphere involvement is almost exclusively limited to the vaginal mucosa and is sometimes responsible for vaginitis (159 publications). It should be noted that the isolation of yeasts from the vaginal mucosa is not systematically pathological and can refer to colonisation [149]. Unusual female genital sphere infection sites were sporadically reported, as in a case of a tubo-ovarian abscess caused by *Candida kefyr* [150] and endometritis caused by *Trichosporon beigelii* [151].

Concerning the male genital sphere, yeast isolation is rarer and often related to glans colonisation (14 publications). Interestingly, it has been shown that yeast colonisation was more frequently observed among uncircumcised versus circumcised men [152,153]. Urethral involvement is rare [154] and one case of orchi-epididymitis post-dissemination of *Geotrichum capitatum* has been reported, but it remains exceptional [155].

#### 3.2.14. Anatomical Sites Rarely Involved

Dental location. Only Ascomycota were reported in the dental location, probably due to the commensal nature of the oral mucosa. Yeasts were mainly found in the dental mycobiota or dental plaque. Two cases of dento-alveolar abscesses due to *Meyerozyma guilliermondii* (anc. *Candida guilliermondii*) [156] and *Magnusiomyces capitatus* (anc. *Trichosporon capitatum*) [157] were found.

Endocrine glands. Only Basidiomycota were reported among endocrine glands with 15 publications reporting *Trichosporon* spp. and 1 publication reporting *Malassezia furfur*. *Trichosporon* species were *Cutaneotrichosporon cutaneum*, *Trichosporon asahii*, *Trichosporon inkin*, and *Trichosporon capitatum*. All the cases were autopsy findings secondary to the disseminated infection.

Breast. Ascomyceta and Basidiomycota have rarely been isolated from this particular site and few infections have been reported. Long-term nipple discharge due to *Pityrosporum orbiculare* (now *Malassezia furfur*) has been described [158]. Interestingly, one case of *Trichosporon beigelii* (now *Cutaneotrichosporon cutaneum*) breast implant infection has been reported in an immunocompetent patient in Thailand [159]. *Saccharomyces cerevisiae* [160,161], *Lodderomyces elongisporus* [162], and *Malassezia globosa* [160] have all been isolated from milk samples.

Placental infection. Only Ascomycota were reported among placental infections. One cases of sepsis with chorioamnionitis caused by *Kluyveromyces marxianus* (formerly *Candida kefyr*) was reported with placental transmission to premature fraternal twins [163]. *Clavispora lusitaniae* was responsible for two cases of chorioamnionitis with foetal infections [164,165]. *Saccharomyces cerevisiae* has been isolated from placental samples [166].

## 4. Discussion

There is increasing clinical interest in less common yeasts implicated in infectious diseases in immunosuppressed patients. The aim of this repertoire was to catalogue as exhaustively as possible the uncommon yeasts identified in humans by culture and molecular biology, whether or not they were associated with histopathological findings.

We opted to exclude some yeast species categorised as “fungal priority pathogens” by the WHO [167]. These include, notably, the five prominent *Candida* species (*Candida albicans*, *C. tropicalis*, *C. glabrata*, *C. parapsilosis*, and *P. kudriavzevii*), along with those from the *Cryptococcus neoformans*/*gattii* complex. This deliberate choice aims to avoid overwhelming the repertoire with publications about these common yeasts, to ensure a more balanced understanding of fungal diversity, and to help uncover valuable information about rarer yeasts that might otherwise remain hidden. We found that 192 less common or rare yeasts, including 127 Ascomycota and 65 Basidiomycota, had been identified in humans. The specific anatomical locations or samples in which these fungi were detected, along with the characteristics of the infections, were clearly delineated. The first publication reporting the isolation of one of the less common yeasts dates back to 1947 and concerns *Pityrosporum ovale* (now *Malassezia furfur*) [168]. To ensure consistency, we chose to stop our analysis on 16 June 2020. The most recently described species in our repertoire is *Rhodotorula toruloides*, isolated from blood culture and identified by sequencing, presenting an emerging agent of bloodstream infection [169].

Systemic location was predominant with a total of 1309 publications. Interestingly, when synonyms and former names are taken into account, *Saccharomyces cerevisiae* is the predominant species (91 publications), followed by *Cutaneotrichosporon cutaneum* (90 publications) and with *Candida auris* in third place (86 publications). However, *Candida auris* is listed as a “fungal priority pathogen” [167], unlike the other two predominant pathogens. *S. cerevisiae* and *Cutaneotrichosporon cutaneum* do not attract as much attention, even though they present real emerging trends.

This fungal repertoire presents a useful tool for diagnostic management. Some less common or rare yeast species may be associated with invasive infections in high-risk patients, and their description could be used to pinpoint them. Considering that the most commonly isolated aetiological agents in mycotic endocarditis are *Candida* spp. and *Aspergillus* spp., there might be a propensity to focus solely on their detection, effectively excluding the possibility of other fungal origins [170]. However, this fungal repertoire highlights the cardiac tropism of *Trichosporon* spp., which may go unnoticed in comparison with much more common species, and must be considered [171]. *Trichosporon* yeasts are opportunistic agents leading to superficial-to-severe infections in at-risk populations [64]. This repertoire emphasises their invasive potential, as they are widely represented in deep-seated localisations. They are some of the less common yeasts most frequently found in the heart and liver. Similarly, they are second only to *Candida* species in systemic localisations, the central nervous system, the pulmonary system, where *Trichosporon beigelii* is the second most common cause of yeast pneumonia, and the digestive system, where they are mostly found in the spleen and involved in peritonitis. We also found some surprising localisations, such as for species of the *Cryptococcus* genus. In addition to the expected locations in the bloodstream, the lungs, the cutaneous system, and the CNS, we found less common *Cryptococcus* species in the ocular region, responsible for keratitis and endophthalmitis. A distinction between Ascomycota and Basidiomycota has also been made for certain rarer localisations. In this way, we saw that endocrine gland damage during dissemination is limited to autopsy findings for Basidiomycota. Similarly, placental damage is limited to Ascomycota and, more specifically, *Candida kefyr* and *Candida lusitaniae*, probably leading to their presence in the genital mucosa, and systematically resulting in foetal damage [163,164,165].

This repertoire also provides an update of the current taxonomy, bringing together the old and new names, taking into account the “one fungus, one name” unification, helping clinicians to identify rare yeast isolations and their synonyms [15,16]. Due to constantly evolving fungal taxonomy, some yeast species may be over- or underestimated, creating a bias, and classifications are bound to evolve. One well-known example is *Candida dubliniensis*, which was identified as *Candida albicans* until 1995 resulting in an underestimation of its involvement in human pathology [19].

One other bias of our study lies in the choice of query following the pattern (fungi_name_or_synonyms [TIAB] OR fungi_name_or_synonyms[OT] OR fungi_name_or_synonyms[MeSH]) AND (“Human”[TIAB] OR “Human”[OT] OR “Humans”[MeSH]). The title, abstract, authors’ keywords, and MeSH descriptors of a PubMed/MEDLINE bibliographic record are particularly important for understanding and grasping the content of an article, as they contain the semantics and main concepts addressed in the article. We choose the MeSH descriptor “Human(s)” as it is a “Check Tag” that is systematically added by the human indexer of the PubMed/MEDLINE database even if the word “human” does not appear in the text (see https://www.nlm.nih.gov/bsd/indexing/training/CHK_010.html accessed on 15 November 2019). However, it is possible that a few articles may not have been identified.

Finally, as for the non-dermatophytic mould repertoire [14], one limitation lies in its temporal scope. Only references which existed in PubMed up until 16 June 2020 were considered, ensuring uniformity across all fungal species. Nonetheless, medical mycology is dynamic, and newly emerging organisms constantly need to be taken into account by clinicians and microbiology laboratories [172]. It will therefore be necessary to update these data regularly.

## Figures and Tables

**Table 1:** Anatomical sites and nosological framework of the different taxa. CNS: central nervous system; ORL: oto-rhino-laryngology; the numbers in the table refer to PMIDs.

	Ascomycota					Basidiomycota						Total
	*Candida* spp.^1^	*Magnusiomyces*/*Saprochaete* clade	Others	*Saccharomyces* spp.	Total	*Cryptococcus* spp.^2^	*Malassezia* spp.	Others	*Rhodotorula* spp.	*Trichosporon* spp.	Total	
**Systemic**	**682**	**87**	**23**	**95**	**887**	**37**	**70**	**9**	**64**	**242**	**422**	**1309**
Anatomical site												
Unspecified	11	2	1		14				3	4	7	21
Blood	664	79	19	90	852	36	70	8	59	220	393	1245
Bone marrow	3	2		1	6				1	7	8	14
Lymph nodes	4	2	3	2	11	1		1	1	7	10	21
Semiology												
Aortitis		1		2	3							3
Vasculitis		1			1					4	4	5
**CNS**	**35**	**4**		**2**	**41**	**19**	**3**	**3**	**12**	**29**	**66**	**107**
Anatomical site												
Unspecified	15	1		1	17	2	1		2	8	13	30
Specimen												
Brain abscess	4	2			6					4	4	10
Semiology												
Encephalitis		1			1							1
Mass				1	1					1	1	2
Meningitis	14				14	13	1	3	9	14	40	54
Meningo-encephalitis	2				2	4	1		1	2	8	10
**Ocular**	**54**	**8**	**1**	**2**	**65**	**9**	**14**	**1**	**6**	**9**	**39**	**104**
Anatomical site												
Unspecified	10	1			11	2	4		1	2	9	20
Conjunctival	3	1			4		2				2	6
Orbital	4				4							4
Specimen												
Lacrimal fluid	5	2			7		1				1	8
Semiology												
Blepharitis							6				6	6
Endophthalmitis	16	1	1		18	1		1	3	5	10	28
Keratitis	16	3		2	21	6	1		2	2	11	32
**Auditory system**	**35**	**1**			**36**	**1**	**11**			**8**	**20**	**56**
Anatomical site											0	
Unspecified	23				23		10			5	15	38
Semiology												
Otomycoses	12	1			13	1	1			3	5	18
**Dental and gums**	**16**	**4**		**4**	**23**							**24**
Anatomical site												
Unspecified	15	2		2	19							19
Specimen												
Abscess	1	1			2							2
Semiology												
Periodontitis		1		2	3							3
**ORL system**	**302**	**21**	**3**	**50**	**376**	**7**	**15**		**7**	**51**	**80**	**456**
Anatomical site												
Unspecified	6			1	7					1	1	8
Nasal	14	1		2	17	2	2			2	6	23
Oesophagus	9	3	1	3	16				1	12	13	29
Oral mucosa	224	11	2	34	271	2	3		6	14	25	296
Pharyngeal	12			1	13	1	1			1	3	16
Rhino-sinusitis	3				3	1	3			2	6	9
Tongue	7	1		2	10		1			2	3	13
Tracheal	22	5		7	34	1	5			17	23	57
Tonsil	5				5							5
**Lung**	**215**	**66**	**13**	**37**	**331**	**23**	**8**	**5**	**5**	**143**	**184**	**515**
Anatomical site												
Unspecified	6	7	1		14	3			3	4	10	24
Lower respiratory tract	68	23	4	17	112	10	5	1		58	74	186
Lymph nodes	1	5		1	7					4	4	11
Mediastinum	1		2	1	4					2	2	6
Parenchymal cavity	2				2					2	2	4
Pleura	15	2	1	4	22	2		2		15	19	41
Upper respiratory tract	108	15	3	11	137	4	1	1	1	38	45	182
Specimen												
Abscess						1				1	2	2
Semiology												
Interstitial pneumonitis		1			1							1
Invasive	2	1	2		5					3	3	8
Pneumonia	12	12		3	27	3	2	1	1	15	22	49
**Breast**			**1**	**2**	**3**		**2**			**1**	**3**	**6**
Anatomical site												
Breast implant										1	1	1
Nipples							1				1	1
Specimen												
Milk			1	2	3		1				1	4
**Heart**	**31**	**4**	**1**	**6**	**42**		**2**		**4**	**40**	**46**	**88**
Anatomical site												
Unspecified	2	1		1	4				1	17	18	22
Myocardium		1			1					5	5	6
Pericardium	4			1	5					4	4	9
Semiology												
Implanted device endocarditis	7			2	9				2	11	13	22
Native valve endocarditis	18	2	1	2	23		2		1	3	6	29
**Enteric**	**155**	**35**	**7**	**60**	**257**	**8**	**15**		**12**	**89**	**124**	**381**
Anatomical site												
Unspecified	73	23	4	40	140	3	6		1	28	38	178
Appendix							1				1	1
Biliary tract	12	2		3	17					2	2	19
Colitis				2	2		1				1	3
Enteritis	1				1							1
Gastric	6	1		2	9		1			5	6	15
Pancreas	1			1	2		1			4	5	7
Spleen	6	5		2	13		2			24	26	39
Specimen												
Abscess	25			2	27							27
Peritoneal fluid	5				5				1		1	6
Semiology												
Cholecystitis	2				2							2
Peritonitis	25	4	3	8	40	5			10	19	34	74
**Liver**	**17**	**8**		**7**	**32**		**1**	**1**	**1**	**37**	**40**	**72**
Anatomical site												
Unspecified	6	5		2	13		1			27	28	41
Specimen												
Abscess	3	1		3	7			1	1	2	4	11
Ascites fluid	7	1		2	10					7	7	17
Semiology												
Hepatitis	1	1			2					1	1	3
**Urinary tract**	**199**	**20**	**1**	**24**	**244**	**3**	**9**		**1**	**108**	**121**	**365**
Anatomical site												
Unspecified	5				5					1	1	6
Bladder	1				1					1	1	2
Kidney	11	3		3	17		1			23	24	41
Prostate gland	1				1							1
Specimen												
Urine	162	14		17	193	3	8		1	58	70	263
Semiology					0							
Mass	5			1	6							6
Pyelonephritis	2	1		1	4					1	1	5
Urinary tract infection	12	2	1	2	17					24	24	41
**Genital**	**110**	**3**		**55**	**168**	**3**	**13**		**4**	**11**	**31**	**199**
Anatomical site												
Unspecified	8	2			10	1	1		4	2	8	18
Endometrium										1	1	1
Epididymis		1			1							1
External genitalia							1			2	3	3
Glans	3			1	4		10				10	14
Ovaries	1				1							1
Vaginal mucosa	98			53	151	2	1			5	8	159
Specimen												
Sperm										1	1	1
Semiology												
Urethral infection				1	1							1
**Osteo-articular system**	**47**	**7**		**2**	**56**	**2**			**4**	**16**	**22**	**78**
Anatomical site												
Unspecified	12				12	1			1	2	4	16
Joint	5	1			6				1	4	5	11
Specimen												
Synovial fluid	5	1			6	1				2	3	9
Semiology												
Arthritis	8	3			11					3	3	14
Mass (including mycetoma)	1				1							1
Osteomyelitis	10	1		2	13				1	3	4	17
Prosthesis-associated Osteitis									1		1	1
Spondylodiscitis	6	1			7					2	2	9
**Skeletal muscles**	**1**				**1**					**1**	**1**	**2**
Anatomical site												
Unspecified	1				1					1	1	2
**Soft tissue**	**4**				**4**					**5**	**5**	**9**
Anatomical site												
Unspecified	4				4					5	5	9
**Skin**	**204**	**30**	**5**	**7**	**246**	**26**	**355**	**5**	**18**	**183**	**587**	**833**
Anatomical site												
Unspecified	10	1		2	13	3			6	8	17	30
Nails	48	1	3	2	54	1	9		5	28	43	97
Subcutaneous	8	1		1	10	2	1	1		10	14	24
Superficial cutaneous	133	25	2	1	161	18	302	2	5	121	448	609
Semiology												
Dermatitis				1	1		43	1			44	45
Mycetoma	1				1			1			1	2
Tinea capitis									1	10	11	11
Tinea cruris	1				1							1
Tinea genitalis										1	1	1
Tinea pedis	2	2			4					1	1	5
Ulcer	1				1	2			1	4	7	8
**Endocrine gland**							**1**			**15**	**16**	**16**
Anatomical site												
Adrenal							1			4	5	5
Thymus										2	2	2
Thyroid										9	9	9
**Placental infection**	**3**			**1**	**4**							**4**
Anatomical site												
Placenta				1	1							1
Semiology												
Chorioamnionitis	3				3							3
**Total**	**2110**	**298**	**55**	**354**	**2816**	**138**	**519**	**24**	**138**	**988**	**1808**	**4624**

^1^ Excluding Candida albicans, *C. tropicalis*, *C. glabrata*, *C. parapsilosis*, and *P. kudriavzevii* (syn. *C. krusei*). ^2^ Excluding *Cryptococcus neoformans* species complex and *Cryptococcus gattii* species complex.

**Table 2:** Details of cardiac sites affected by native valve endocarditis and associated species.

Name *	Tricuspid Valve	Mitral Valve	Aortic Valve	Endocardium	Pulmonary Valve	Atrium	Ventricle	ND
*Blastoschizomyces capitatus*								1 [90]
*Candida colliculosa*	1 [91]							
*Candida dubliniensis*	2 [92,93]		1 [94]					
*Candida guilliermondii*					1 [95]			
*Candida kefyr*		1 [96]						
*Candida lusitaniae*			2 [97,98]					
*Candida mycoderma*							1 [99]	
*Candida sake*		1 [100]	1 [101]					
*Candida zeylanoides*		1 [102]						
*Geotrichum clavatum*	1 [103]	1 [103]						
*Hansenula anomala*			1 [104]					
*Kodamaea ohmeri*	1 [105]							1 [106]
*Lodderomyces elongisporus*			1 [37]					
*Malassezia furfur*	1 [107]	1 [107]						1 [108]
*Pichia fabianii*			1 [109]					
*Pichia ohmeri*		1 [110]						
*Rhodotorula pilimanae*		1 [111]	1 [111]					
*Saccharomyces cerevisiae*		1 [112]				1 [113]		
*Trichosporon asahii*		1 [114]	1 [114]					
*Trichosporon beigelii*	1 [115]			1 [115]			1 [115]	
*Trichosporon cutaneum*			1 [116]					
*Yarrowia lipolytica*								1 [117]
Total	7	10	11	1	1	1	2	4

ND: Not determined. * The species name in the table is that found in the cited publication.

## Data Availability

Data are contained within the article.

## Supplementary Materials

The following supporting information can be downloaded at https://www.mdpi.com/article/10.3390/jof9111099/s1, Table S1: Number of publications found by anatomical site and species for Ascomycota division. Several anatomical sites of isolation could be found in the same publication (PMID); Table S2: Number of publications found by anatomical site and species for the Basidiomycota yeast division. In the same publication (PMID), several anatomical sites of isolation could be found.
