# Supplementary material for: A Repertoire of the Less Common Clinical Yeasts
Source: J Fungi (Basel). 2023 Nov 11;9(11):1099. doi: 10.3390/jof9111099 (PMC10671991; doi:10.3390/jof9111099)
Supplement: Supplementary file 1 [file jof-09-01099-s001.zip › jof-2680506-supplementary.pdf]

Journal of Fungi

MDPI

Table S1. Number of publications found by anatomical site and species for Ascomycota division. Several anatomical sites of isolation could be found in the same publication (PMID).

Name	Current name	Year of first publication	Year of last publication	Systemic	CNS	Eye	Ear	Dental and gums	ORL system	Lung	Breast	Heart	Gut	Liver	Urinary tract	Genitalia	Osteo-articular	Skeletal muscles	Soft tissue	Skin	Endocrine glands	Placenta	Total
***Candida* spp.**				**669**	**35**	**50**	**33**	**16**	**279**	**166**		**31**	**131**	**16**	**183**	**110**	**44**	**1**	**4**	**183**		**3**	**195 4**
Candida allociferrii	*Blastobotrys allociferrii*	2015	2015			1																	1
Candida auris	Under reclassification	2009	2020	86	7	6	21		22	31		1	25		48	2	14		1	46			310
Candida blankii	Under reclassification	2018	2020	3																			3
Candida bracarensis	*Nakaseomyces bracarensis*	2009	2019	2						1			1			2							6
Candida colliculosa	*Torulaspora delbrueckii*	2003	2016									1				1				1			3
Candida conglobata		2017	2017	1																			1
Candida diddensiae		2020	2020	1																			1
Candida dubliniensis	Under reclassification	1995	2020	60	8	7		10	130	35		5	11	4	20	28	4			9			331
Candida duobushaemulonii		2012	2019	4						1						1	1		1	5			13
Candida ernobii	*Nakazawaea ernobii*	2013	2014	1																			1
Candida etchellsii		2014	2014																	1			1
Candida galli	*Yarrowia galli*	2014	2020																	3			3
Candida haemulonii		1991	2020	14									2		1	1	3		2	7			30
Candida humicola	*Vanrija humicola*	1971	1998		1	2							1		1					1			6

Candida inconspicua	Under reclassification	1997	2019	6					2	7			2	1	1	6				3			28
Torulopsis inconspicua	*Candida inconspicua*	1973	1999	1													1						2
Candida intermedia		1954	2018	4						1						2							7
Candida magnoliae	*Starmerella magnoliae*	1995	2020	1		1		1															3
Candida membranaefaciens		2011	2017	2(4)																			2(4)
Candida membranifaciens	*Candida membranaefaciens*	2011	2017	2																			2
Candida mengyuniae	*Cyberlindnera mengyuniae*	2011	2011	1																			1
Candida mesorugosa	*Diutina mesorugosa*	2013	2019	2									1		1					1			5
Candida metapsilosis		2005	2020	31			2		3	4			3		5	6				16			70
Candida mycoderma	*Candida vini*	1958	2016			1				3		1	1	1	1								8
Candida nivariensis	*Nakaseomyces nivariensis*	2005	2020	11					4	7			2	2	6	6				1			39
Candida orthopsilosis	Under reclassification	2005	2020	35		3	2		5	3			2		10	5	1			9			75
Candida palmioleophila	Under reclassification	1999	2020	3		1			1						1								6
Candida pararugosa	*Diutina pararugosa*	2004	2019	3					2											1			6
Candida pintolopesii	*Kazachstania pintolopesii*	1987	1987						1														1
Candida pseudoaaseri		2011	2011	1																			1
Candida pseudohaemulonii	Under reclassification	2009	2012	4											1								5
Candida pseudohumilis	*Kazachstania pseudohumilis*	2009	2009						1														1
Candida pseudorugosa	*Diutina pseudorugosa*	2012	2013	1						1													2
Candida quercitrusa	*Kurtzmaniella quercitrusa*	2014	2015	2																			2
Candida rugosa	*Diutina rugosa*	1985	2019	19	1				3	4			3		2	1	1			3			37
Candida sake	Under reclassification	1998	2011	1					2	1		2	1		1	1	1						10
Candida spencermartinsiae		2018	2018						1														1
Candida sphaerica	*Kluyveromyces lactis*	2010	2014	1					1														2
Candida stellimalicola		2018	2018										1										1
Candida tunisiensis	Under reclassification	2013	2013						1														1

Candida viswanathii	Under reclassification	1976	2015	1	1															1			3
Candida wangnamkhiaoensis		2016	2016	1																			1
Candida zeylanoides	Under reclassification	1987	2019	3								1	1			2	1			2			10
Clavispora lusitaniae		1990	2020	7 (78)	2(6)	(4)	(1)	(1)	1 (18)	2 (20)		(4)	2 (22)	(3)	2 (34)	(16)	(6)			2 (13)		(2)	18 (231)
Candida lusitaniae	*Clavispora lusitaniae*	1979	2020	74	4	4	1	1	17	18		4	20	3	32	16	6			11		2	213
Cyberlindnera fabianii		2016	2019	4 (11)			(1)		(2)	(3)		(1)	1(3)		(4)	(1)				(1)			5 (27)
Candida fabianii	*Cyberlindnera fabianii*	2006	2016	4			1		1	1			1		2	1				1			12
Hansenula fabianii	*Cyberlindnera fabianii*	1990	1990												1								1
Lindnera fabianii	*Cyberlindnera fabianii*	2012	2014	3					1				1		1								6
Pichia fabianii	*Cyberlindnera fabianii*	2006	2015	4						2		1											7
Cyberlindnera jadinii		2016	2019	(6)		(1)				(1)					(1)	1(1)				1(1)			2 (11)
Candida utilis	*Cyberlindnera jadinii*	1988	2018	6		1									1					1			9
Torula utilis	*Cyberlindnera jadinii*	1965	1965							1													1
Debaryomyces hansenii		1982	2017	1 (20)	(2)	(4)	(1)	(1)	1(8)	2(4)		(1)	3 (10)	(1)	1(3)	(6)	1(1)	(1)		2 (11)			11 (74)
Debaryomyces kloeckeri	*Debaryomyces hansenii*	1960	1960	1														1		1			3
Candida famata	*Debaryomyces hansenii*	1986	2020	17	2	3	1	1	7	2		1	7	1	2	6				5			55
Torulopsis candida	*Debaryomyces hansenii*	1969	1991	1		1														3			5
(*Diutina catenulata*)		NA	NA	(2)																(1)			(3)
Candida catenulate	*Diutina catenulata*	1999	2018	2																			2
Candida ravautii	*Diutina catenulata*	1977	1977																	1			1
(*Kazachstania exigua*)		NA	NA													(1)	(1)						(2)
Candida holmii	*Kazachstania exigua*	2007	2007													1							1

Torulopsis holmii	*Kazachstania exigua*	1983	1983														1						1
Kluyveromyces marxianus		2014	2020	2 (29)	(1)	(1)	(1)		2 (23)	1(7)		(1)	(10)		1 (18)	(9)				(3)		(1)	6 (104)
Candida kefyr	*Kluyveromyces marxianus*	1992	2020	22			1		19	6		1	7		14	8				3		1	82
Candida pseudotropicalis	*Kluyveromyces marxianus*	1955	2014	5	1	1			2				3		3	1							16
Kodamaea ohmeri		2004	2020	23 (31)		1			4(5)	2		3(5)	2(3)		(1)					6(8)			41 (56)
Pichia ohmeri	*Kodamaea ohmeri*	1998	2013	8					1			2	1		1					2			15
Metschnikowia pulcherrima		1972	1998	1(6)						1					1(2)					(1)			3 (10)
Candida pulcherrima	*Metschnikowia pulcherrima*	1972	2018	5											1					1			7
Meyerozyma caribbica		2019	2019	(8)		(1)			(2)	1(3)			(2)		(1)		(1)						1(18)
Candida fermentati	*Meyerozyma caribbica*	2008	2018	8		1			1	2			2		1		1						16
Pichia caribbica	*Meyerozyma caribbica*	2013	2013						1														1
Meyerozyma guilliermondii		2011	2021	6 (66)	(6)	(5)	(2)	(2)	(26)	2 (13)		(5)	1(14)	(3)	1 (13)	(11)	(4)			1 (22)			11 (192)
Candida guilliermondii	*Meyerozyma guilliermondii*	1970	2020	58	6	5	2	2	25	9		5	12	3	11	11	4			21			174
Pichia guilliermondii	*Meyerozyma guilliermondii*	2008	2016	2					1	2			1		1								7
Pichia norvegensis		1976	1976	(10)				(1)	(5)	(3)		(1)	(5)	(1)	(4)	1				(1)			1 (32)
Candida norvegensis	*Pichia norvegensis*	1990	2018	10				1	5	3		1	5	1	4					1			31
Pichia fermentans		2015	2015	(4)						1							(1)						1(6)
Candida lambica	*Pichia fermentans*	1995	2012	4													1						5
Stephanoascus ciferrii		2010	2018	(3)		1(2)	1(2)			1(2)										(4)			3

																							(13)
Candida ciferrii	*Stephanoascus ciferrii*	1983	2017	3		1	1			1										4			10
Wickerhamomyces anomalus		2012	2017	5 (56)	(2)	2(6)			1(6)	(3)		(1)	(4)		(3)		1(2)			(3)			9 (86)
Candida pelliculosa	*Wickerhamomyces anomalus*	1991	2018	14	1	2			2														19
Hansenula anomala	*Wickerhamomyces anomalus*	1958	2010	27	1	1			1	2		1	2		3		1			1			40
Pichia anomala	*Wickerhamomyces anomalus*	1999	2014	10		1			2	1			2							2			18
Yarrowia lipolytica		1985	2020	11 (23)		1(3)			(5)	1(3)		1	(1)			(1)	1			1(3)			16 (41)
Candida lipolytica	*Yarrowia lipolytica*	1976	2017	12		2			5	2			1			1				2			25
Zygoascus hellenicus		2004	2004	1																			1
Candida hellenica	*Zygoascus hellenicus*	2004	2011							1													1
***Magnusiomyces/Saprochaete* clade**				**95**	**4**	**7**	**2**	**4**	**25**	**48**		**6**	**39**	**9**	**19**	**3**	**6**			**30**	**1**		**298**
Geotrichum candidum		1953	2019	11	1	4 (5)	1	2	9	7		1	15 (16)	1	3	2	1			15			73 (75)
Galactomyces geotrichum	*Geotrichum candidum*	2012	2017			1							1										2
Magnusiomyces capitatus		2016	2019	4 (73)	(3)	2	(1)	1(2)	2 (15)	4 (37)		(4)	3 (19)	(6)	1 (16)	(1)	(5)			2 (12)	(1)		19 (197)
Blastoschizomyces capitatus	*Magnusiomyces capitatus*	1990	2018	5	1				1	4		1	1	1	1		1			1			17
Dipodascus capitatus	*Magnusiomyces capitatus*	2004	2017	5	1				1	3					1		1						12
Geotrichum capitatum	*Magnusiomyces capitatus*	1989	2020	28	1				4	14			4	2	6	1	1			3			64
Saprochaete capitata	*Magnusiomyces capitatus*	2011	2020	20					2	7			4		3		1			4			41

Trichosporon capitatum	*Magnusiomyces capitatus*	1965	2012	11			1	1	5	5		3	7	3	4		1			2	1		44
(*Magnusiomyces clavatus*)		NA	NA	(11)					(1)	(4)		(1)	(4)	(2)						(3)			(26)
Geotrichum clavatum	*Magnusiomyces clavatus*	2014	2019	5					1			1	2	1									10
Saprochaete clavata	*Magnusiomyces clavatus*	2016	2019	6						4			2	1						3			16
***Saccharomyces* spp.**				**93**	**2**	**2**		**4**	**44**	**26**	**2**	**5**	**52**	**7**	**19**	**55**	**2**			**7**		**1**	**321**
Saccharomyces kluyveri	*Lachancea kluyveri*	1998	2016	2	1					1			1	1	1								7
Saccharomyces cerevisiae		1970	2020	76 (91)	1	2		4	43	25	2	5	50 (51)	6	18	55	2			7		1	297 (313)
Saccharomyces boulardii	*Saccharomyces cerevisiae*	1991	2012	14									1										15
Saccharomyces cerevisiae var. boulardii	*Saccharomyces cerevisiae*	2020	2020	1																			1
Saccharomycopsis capsularis		2003	2003						1														1
**Others**				**21**		**1**			**3**	**8**	**1**	**1**	**7**		**1**	**1**				**5**			**49**
Arxula adeninivorans	*Blastobotrys adeninivorans*	2015	2015	1						1													2
Blastobotrys proliferans		2007	2007										1										1
Debaryomyces nepalensis		2019	2019	1						1													2
Diutina neorugosa		2019	2019																	1			1
(*Ogatea polymorpha*)		NA	NA	(2)						(2)													(4)
Hansenela polymorpha	*Ogataea polymorpha*	1980	1980	1						1													2
Hansenula polymorpha	*Ogataea polymorpha*	1980	1980	1						1													2
Hanseniaspora opuntiae		2018	2018	1																			1
Hanseniaspora uvarum		1998	2015										1(2)							2(3)			3(5)
Kloeckera apiculata	*Hanseniaspora uvarum*	1998	1998										1							1			2
Hanseniaspora valbyensis		1960	1960										1							1			2
Issatchenkia occidentalis		2006	2006						1														1
Pichia terricola	*Issatchenkia terricola*	2019	2019												1								1

Kluyveromyces fragilis		1980	1980							1													1
Lachancea fermentati		2014	2014	1						1			1										3
Kluyveromyces waltii	*Lachancea waltii*	2012	2012										1										1
Lodderomyces elongisporus		2008	2019	8							1	1											10
Metschnikowia sinensis		2013	2013							1													1
Pichia farinosa	*Millerozyma farinosa*	1989	2015	3					1														4
Monilia sitophila		1996	1996										1										1
Pichia kluyveri		2018	2018						1														1
Schwanniomyces etchellsii		2016	2016	1																			1
Sporopachydermia cereana		2011	2017	3																			3
Torulopsis magnoliae		1994	1994			1																	1
Torulopsis pintolopesii		1989	1989							1													1
**Total**				**878**	**41**	**60**	**35**	**24**	**351**	**248**	**3**	**43**	**229**	**32**	**222**	**169**	**52**	**1**	**4**	**225**	**1**	**4**	**262<br>2**

CNS: Central nervous system; ORL: oto-rhino-laryngology; NA: Not applicable; (n): number of publications corresponding to all isolates from the same species on the basis of their current taxonomic; (*species name*): yeast not found under this name corresponds to the current taxonomy, according to our algorithm.

Table S2. Number of publications found by anatomical site and species for the Basidiomycota yeasts division. In the same publication (PMID) several anatomical sites of isolation could be found.

Name	Current name	Year of first publication	Year of last publication	Systemic	CNS	Eye	Ear	Dental and gums	ORL system	Lung	Breast	Heart	Gut	Liver	Urinary tract	Genitalia	Osteo-articular	Skeletal muscles	Soft tissue	Skin	Endocrine glands	Placenta	Total
***Cryptococcus* spp.**				**37**	**18**	**9**	**1**		**7**	**18**			**8**		**3**	**3**	**2**			**23**			**129**
Cryptococcus adeliensis	*Naganishia adeliensis*	2004	2005		1				1	1													3
Cryptococcus arboriformis	*Cutaneotrichosporon arboriforme*	2007	2015	1									1		1								3
Cryptococcus chernovii	*Filobasidium chernovii*	2011	2011						1														1
Cryptococcus curvatus	*Cutaneotrichosporon curvatus*	1995	2019	1	3	1										1							6
Cryptococcus flavescens	*Papiliotrema flavescens*	2004	2004		1																		1
Cryptococcus glabratus	*Nakaseomyces glabratus*	1950	1950													1							1
Cryptococcus humicola	*Vanrija humicola*	2001	2010	1					1	2										2			6
Cryptococcus liquefaciens	*Naganishia liquefaciens*	2014	2015	1	1																		2
Cryptococcus luteolus	*Hannaella luteola*	1955	2014							1							1						2
Cryptococcus macerans	*Cystofilobasidium macerans*	1997	1997		1																		1
Cryptococcus magnus	*Filobasidium magnum*	2011	2011						1														1
Cryptococcus terreus	*Solicoccozyma terreus*	1994	1994	1																			1
Naganishia albida		2017	2020	1	(3)	(5)	1		(2)	(7)			(2)		(1)	(1)	(1)			1			3

				(13)																(12)			(48)
Cryptococcus albidus	*Naganishia albida*	1972	2020	12	3	5			2	7			2		1	1	1			11			45
Papiliotrema laurentii		2019	2019	1 (19)	(8)	(3)			(1)	(7)			(5)		(1)					(9)			1 (53)
Cryptococcus laurentii	*Papiliotrema laurentii*	1976	2020	18	8	3			1	7			5		1					9			52
***Malassezia* spp.**				**70**	**3**	**13**	**11**		**13**	**6**	**2**	**2**	**13**	**1**	**9**	**13**				**316**	**1**		**473**
Malassezia arunalokei		2016	2016																	1			1
Malassezia dermatis		2002	2018																	6			6
Malassezia furfur		1968	2020	51	2	4(9)	2(5)		4	5	(1)	2	5	1	6(7)	4(5)				89 (161)	1		176 (259)
Pityrosporum orbiculare	*Malassezia furfur*	1963	2005			3	1				1				1	1				27			34
Pityrosporum ovale	*Malassezia furfur*	1947	2008			2	2													45			49
Malassezia globosa		2000	2020						1		1					3				47			52
Malassezia japonica		2003	2015																	3			3
Malassezia obtusa		2005	2016													1				7			8
Malassezia pachydermatis		1987	2020	16	1	2	2		3	1			2		2	1				18			48
Malassezia restricta		2002	2020			1	1		4				6							32			44
Malassezia slooffiae		2005	2017				2									2				10			14
Malassezia sympodialis		1998	2020	3		1	1		1							1				31			38
***Rhodotorula* spp.**				**64**	**12**	**8**			**7**	**5**		**4**	**12**	**1**	**1**	**4**	**4**			**18**			**140**
Cystobasidium minutum		2020	2020	2(5)		(1)									(1)		(2)			(1)			2 (10)
Rhodotorula minuta	*Cystobasidium minutum*	1991	2020	3		1									1		2			1			8

Rhodotorula glutinis		1980	2015	12	4	3				1			2			1							23
Rhodotorula mucilaginosa		1992	2019	27 (46)	4(8)	1(4)			1(7)	1(4)		2(3)	5 (10)	1		1(3)	2			12 (17)			57 (105)
Rhodotorula rubra	*Rhodotorula mucilaginosa*	1975	2014	19	4	3			6	3		1	5			2				5			48
Rhodotorula pilimanae		1975	1975									1											1
Rhodotorula toruloides		2020	2020	1																			1
***Trichosporon* spp.**				**218**	**28**	**8**	**7**		**38**	**92**	**1**	**33**	**63**	**34**	**84**	**10**	**12**	**1**	**5**	**164**	**11**		**809**
Cutaneotrichosporon debeurmannianum		2018	2020	1											1					1(2)			3(4)
Trichosporon debeurmannianum	*Cutaneotrichosporon debeurmannianum*	2016	2016																	1			1
Endomycopsis mali	*Trichosporon faecale*	1959	1959										1										1
Trichosporon asahii		1996	2020	86	10	3	5		11	27		3	17	11	46	2	5		4	55	1		286
Trichosporon asteroides		2002	2019	6						1							1			1			9
(*Cutaneotrichosporon cutaneum*)		NA	NA	(90)	(14)	(5)	(2)		(21)	(40)	(1)	(26)	(35)	(19)	(31)	(6)	(4)	(1)	(1)	(55)	(9)		(376)
Trichosporon beigelii	*Cutaneotrichosporon cutaneum*	1968	2007	72	10	2	1		17	34	1	22	27	16	27	5	4	1	1	53	8		301
Trichosporon cutaneum	*Cutaneotrichosporon cutaneum*	1955	2017	18	4	3	1		3	6		4	8	3	4	1				16	1		72
Trichosporon cutaneum	*Cutaneotrichosporon cutaneum*	1964	1994						1											1			2
Trichosporon coremiiforme		2009	2020	3									1										4
Trichosporon dohaense		2009	2019	3																2			5
Trichosporon inkin		1996	2019	8	3				2	6		3	5	1	3	1	2			18	1		53
Trichosporon japonicum		2008	2019	1						2													3

Trichosporon jirovecii	*Cutaneotrichosporon jirovecii*	2012	2019											1		1							2
Trichosporon loubieri	*Apiotrichum loubieri*	2003	2019	3						1			1	1	2					2			10
Trichosporon montevideense	*Apiotrichum montevideense*	2019	2020										1	1						1			3
Trichosporon mucoides	*Cutaneotrichosporon mucoides*	1996	2019	8	1				3	2			1							8			23
Trichosporon mycotoxinivorans	*Apiotrichum mycotoxinivorans*	2009	2020	3					1	9		1	1							1			16
Trichosporon ovoides		2008	2019	1											1					4			6
Trichosporon pullulans	*Guehomyces pullulans*	1988	2006	5						4													9
**Others**				**9**	**3**	**1**				**5**				**1**						**4**			**23**
Apiotrichum veenhuisii		2019	2019																	1			1
Fereydounia khargensis		2016	2016	1						1													2
Pseudozyma alboarmeniaca		2014	2014	1																			1
Pseudozyma aphidis	*Moesziomyces aphidis*	2008	2016	4						2										1			7
Pseudozyma siamensis	*Ustilago siamensis*	2014	2014	1																			1
Sporobolomyces holsaticus	*Sporobolomyces johnsonii*	1984	1984																	1			1
Sporobolomyces roseus		2012	2012		1																		1
Sporobolomyces salmonicolor		1976	2015	2	2	1																	5
Sporidiobolus salmonicolor	*Sporobolomyces salmonicolor*	2015	2015							1				(1)									1(2)
Sterigmatomyces halophilus		2019	2019											1									1
Wallemia sebi	*Wallemia mellicola*	2008	2016							1										1			2
**Total**				**398**	**64**	**39**	**19**		**65**	**126**	**3**	**39**	**96**	**37**	**97**	**30**	**18**	**1**	**5**	**525**	**12**		**1574**

CNS: Central nervous system; ORL: oto-rhino-laryngology; (n): number of publications corresponding to all isolates from the same species on the basis of their current taxonomic
